# Reference Gene Selection for qRT-PCR Normalization Analysis in kenaf (*Hibiscus cannabinus* L.) under Abiotic Stress and Hormonal Stimuli

**DOI:** 10.3389/fpls.2017.00771

**Published:** 2017-05-12

**Authors:** Xiaoping Niu, Meixia Chen, Xinyu Huang, Huihuang Chen, Aifen Tao, Jiantang Xu, Jianmin Qi

**Affiliations:** ^1^State Key Laboratory of Ecological Pest Control for Fujian and Taiwan Crops, Key Laboratory for Genetics, Breeding and Multiple Utilization of Crops, Fujian Agriculture and Forestry UniversityFuzhou, China; ^2^College of Life Science, Ningde Normal UniversityNingde, China; ^3^College of Life Science, Fujian Agriculture and Forestry UniversityFuzhou, China

**Keywords:** reference gene, kenaf (*Hibiscus cannabinus* L.), abiotic stress, hormonal stimulus, gene expression

## Abstract

Kenaf (*Hibiscus cannabinus* L.), an environmental friendly and economic fiber crop, has a certain tolerance to abiotic stresses. Identification of reliable reference genes for transcript normalization of stress responsive genes expression by quantitative real-time PCR (qRT-PCR) is important for exploring the molecular mechanisms of plants response to abiotic stresses. In this study, nine candidate reference genes were cloned, and their expression stabilities were assessed in 132 abiotic stress and hormonal stimuli samples of kenaf using geNorm, NormFinder, and BestKeeper algorithms. Results revealed that *HcPP2A* (*Protein phosphatase 2A*) and *HcACT7* (*Actin 7*) were the optimum reference genes across all samples; *HcUBC* (*Ubiquitin-conjugating enzyme like protein*) was the worst reference gene for transcript normalization. The reliability of the selected reference genes was further confirmed by evaluating the expression profile of *HcWRKY28* gene at different stress durations. This work will benefit future studies on discovery of stress-tolerance genes and stress-signaling pathways in this important fiber crop.

## Introduction

Being sessile, plants have to adapt and survive in various unfavorable environmental conditions. Water deficiency, excess salinity and extreme temperatures are major abiotic stresses that can occur at multiple stages of plant development, challenging production, and productivity of crop plants (Bray et al., 2000; Mahajan and Tuteja, 2005). In fact, soil desertification and salinization are rapidly increasing on a global scale, declining average yields for most major crop plants by more than 50% (Bartels and Sunkar, 2005). In response to these stress factors, plants can perceive abiotic stresses, and develop diverse signaling pathways comprising the regulation network of protein-protein reactions, protein-DNA interaction to combat or tolerate stresses. These well-known signals pathways including abscisic acid (ABA), ethylene (ET), jasmonic acid (JA), and salicylic acid (SA) pathways, which play vital roles in the plant developmental regulation process, and abiotic or biotic stresses alleviation (Mahajan and Tuteja, 2005; Bari and Jones, 2009). Therefore, elucidating the molecular mechanism of plant tolerance to adverse conditions is of fundamental importance to crop improvement.

Kenaf (*Hibiscus cannabinus* L.), belonging to the Malvaceae family, is an industrial crop holding high cellulosic fiber content, predominantly grown in Asia and Africa (Ayadi et al., 2011; Niu et al., 2015a). Kenaf is an appealing fiber source for the paper manufacture, owing to its environmentally friendly nature, such as biodegradability, renewability, and low energy consumption (Bhardwaj et al., 2005; Villar et al., 2009; Niu et al., 2015a). It has also a great potential for utilization in oil absorption, animal feed, and value-added industrial products (Ayadi et al., 2011; Niu et al., 2015a). Moreover, it is reported that kenaf could survive in areas with deficient water and well adapt to diverse adverse stresses, such as high salinity, drought and extreme temperature (Curtis and Läuchli, 1985; Francois et al., 1992; Banuelos et al., 2002). Thus, it is of fundamental importance to explicate the mechanism of fiber development and stress adaptation of *Hibiscus cannabinus*. However, limited genome sequence information is available, which greatly hinders kenaf functional gene discovery, expression profiling, and functional analysis, ultimately resulting in a slow improving advancement of kenaf fibers. Based on gene expression analysis, several stress response genes were recently identified and validated, which provides us a better reference to study the gene functions (Chuaqui et al., 2002; Zhu, 2002; Rasmussen et al., 2013). In this respect, qRT-PCR, a widely used high-throughout technique, was applied for validating microarray results and transcriptional expression of target genes, due to its high specificity, sensitivity and reproducibility (Bustin, 2002). To obtain an accurate expression data, one or more reference genes are introduced as normalization factors to lessen the variance. However, it is reported that unstable reference gene(s) used as normalization factor can lead to false inferences or misinterpretations of quantification results (Gutierrez et al., 2008; Guenin et al., 2009). Therefore, the stability of reference gene(s) should be systematically evaluated across different varieties, tissue samples, experimental treatments, and developmental stages prior an application in qRT-PCR analysis (Bustin, 2002; Guenin et al., 2009).

Currently, several studies have been performed in various fiber crops for selecting the stable reference genes across different tissue samples, developmental stages and abiotic/biotic stress conditions (Artico et al., 2010; Huis et al., 2010; Niu et al., 2015a,b). However, there is no systematic study performed in any fiber crop for reliable reference gene(s) selection under different hormonal stimuli and abiotic stresses, which limits further the functional gene studies and transcriptomic analysis.

Taken the need for normalization analysis into consideration, the present study was performed to identify and evaluate 9 candidate reference genes: *18S rRNA* (*18S ribosomal RNA*), *ACT7* (*Actin 7*), *UBC* (*Ubiquitin-conjugating enzyme like protein*), *PTB* (*Polypyrimidine tract-binding protein*), *PP2A* (*Protein phosphatase 2A*), *TUB8* (*Beta-tubulin 8*), *MZA* (*Clathrin adaptor complexes medium subunit family protein*), *RAN* (*Ras-related small GTP-binding protein*), and *EF1*α (*Elongation factor 1-alpha*) in kenaf exposed to different abiotic stresses (salinity, drought and cold) and hormonal stimuli (ABA, ET, JA, and SA) at various stress durations. The expression stability of candidate reference genes was assessed by three statistical algorithms, namely, geNorm (Vandesompele et al., 2002), NormFinder (Andersen et al., 2004), and BestKeeper (Pfaffl et al., 2004). Furthermore, the *HcWRKY28* gene of *H. cannabinus* was used to validate the reliability of the selected reference genes under different conditions.

## Materials and methods

### Plant materials and treatments

Three kenaf varieties, Fuhong992, Fuhong952, and GV42, were used for stress treatments. Seeds were germinated on filter paper saturated with water, and seedlings were grown in nutritional soil in an artificial climate chamber (28°C, 16/8 h light/darkness; light intensity 300 μmol·m^−2^·s^−1^; relative humidity 50 ± 10%). For each variety, three 5-leaf-stage seedlings from the same experimental treatments were collected for 3 replicates. For the salinity and drought treatments, the details refer to the method of Niu et al. (2016). Cold treatment was performed by exposing seedlings to 4°C in light and harvested at the same time points. For the hormone treatments, leaves were sprayed with 0.3 mM abscisic acid (ABA treatment), 5.0 mM ethylene (ET treatment), 0.1 mM jasmonic acid (JA treatment), and 0.2 mM salicylic acid (SA treatment) and then harvested at the same time points above. All samples were harvested from three biological replicates in different pots, giving a total of 132 samples comprised of 57 abiotic and 75 hormone treatment samples for each variety. All samples were frozen in liquid nitrogen and stored at −80°C.

### Total RNA isolation and cDNA synthesis

The OMEGA isolation kit (R6827-01, Omega Bio-tech, USA) was used for total RNA isolation. The concentration and purity of each RNA sample was estimated by a NanoDrop ND 2000 spectrophotometer (NanoDrop, Thermo Scientific). RNAs with an OD260/OD280 value between 1.9 and 2.1, and OD260/OD230 > 2.0 were used for further analyses. The first-strand cDNA was synthesized using the PrimeScript® RT reagent kit (TaKaRa, Japan) and stored at –20°C for further use.

### Primer design and PCR products verification

Nine candidate reference genes, 18S *rRNA, ACT7, UBC, PTB, PP2A, TUB8, MZA, RAN*, and *EF1*α, were screened from the *Arabidopsis* database. The potential homologs were identified by querying from the kenaf expressed sequence tags (ESTs) database and the kenaf transcriptome database (SRP060459) (Zhang et al., 2015). Based on these potential reference gene sequences, primers were designed for detection and cloning of reference genes using Primer v6. The detailed information of primers, such as GC content, melting temperatures, primer lengths, and amplicon lengths, were presented in Table 1. Cloning information was provided in Table S1. The specificity of PCR products were confirmed by agarose gel electrophoresis and sequencing.

**Table 1:** **Characteristics of nine candidate reference genes in *H. cannabinus* and parameters derived from qRT-PCR analysis**.

**Gene**	**Gene function**	**Accession number**	**Primer sequence F/R (5'-3')**	**Product (bp)**	**Efficiency (%)**	**R2**	**Average Ct**	**SD**	**CV (%)**
*Hc18S rRNA*	18S ribosomal RNA	FJ527607	CTACGTCCCTGCCCTTTGTA	175	104.1	0.9949	17.98	2.25	12.5
			GGTTCACCTACGGAAACCTTG						
*HcACT7*	Actin 7	KY382441	TTGCAGACCGTATGAGCAAG	166	105.4	0.9967	21.92	2.35	10.7
			ATCCTCCGATCCAGACACTG						
*HcUBC*	Ubiquitin-conjugating enzyme like protein	KY382442	CTGCCATCTCCTTTTTCAGC	150	118.6	0.9981	24.66	2.14	8.7
			CGAGTGTCCGTTTTCATTCA						
*HcPTB*	Polypyrimidine tract-binding protein	KY382443	GGTTACCATTGAGGGTGTGG	158	109.4	0.9993	28.89	1.93	6.7
			GTGCACAAAACCAAATGCAG						
*HcPP2A*	Protein phosphatase 2A	KY382444	GATCCTTGTGGAGGAGTGGA	201	108.9	0.9985	29.14	1.83	6.2
			GCGAAACAGTTCGACGAGAT						
*HcTUB8*	Beta-tubulin 8	KY382445	AATGCTTGCTGGGAGCTTTA	213	105.1	0.9992	30.62	2.35	7.6
			GTGGAATAACTGGCGGTACG						
*HcMZA*	Clathrin adaptor complexes medium subunit family protein	KY382446	CCGTCAGACAGATTGGAGGT	154	106.3	0.9949	34.04	1.69	4.9
			AAAGCAACAGCCTCAACGAC						
*HcRAN*	Ras-related small GTP-binding protein	KY382447	GCCATGCCGATAAGAACATT	167	97.13	0.9997	32.92	2.46	7.4
			GTGAAGGCAGTCTCCCACAT						
*HcEF1α*	Elongation factor 1-alpha	KY382448	TCCCCATCTCTGGTTTTGAG	130	113.8	0.9960	23.33	2.24	9.6
			CTTGGGCTCATTGATCTGGT						

### qRT-PCR assay

qRT-PCR was performed in 96-well plates by using a Applied Biosystems 7500 Real-Time PCR System (Applied Biosystems, USA). cDNA was amplified by using SYBR® Premix *Ex Taq* (Tli RNaseH Plus, TaKaRa, Japan), and a final volume of 20 μl reaction contains 10 μl 2 × SYBR Premix *Ex Taq*, 2 μl cDNA template, 0.4 μl each amplification primer, 0.4 μl ROX Reference Dye II, and 6.8 μl dH_2_O. Thermal cycling was performed with an initial step of 95°C for 30 s, 40 cycles at 95°C for 5 s, and 60°C for 34 s. The final melting curve was obtained from 60° to 95°C to verify primer specificity. All assays were carried out in three technical and biological replicates with template-free negative controls being performed in parallel. The amplification efficiency (E) and correlation coefficient (R^2^) were determined by the standard curves, based on the 10-fold cDNA diluted series.

### Data analysis

Three available statistical algorithms, geNorm, NormFinder, and BestKeeper were used for evaluating the expression stability of each candidate gene. All analyses were conducted following the operation instructions. The raw Ct values of all reference genes were transformed into the required data format. The relative quantities were calculated according to the formula: 2^−ΔCt^, where ΔCt = corresponding Ct—minimum Ct. A comprehensive ranking of reference genes was generated as suggested by Niu et al. (2015a,b). Statistical analysis of gene expression data were performed using the software SPSS 22.0 (SPSS Inc., USA).

## Results

### Cloning and detection of candidate reference genes

On the basis of the homology analysis with *Arabidopsis* genes, 9 candidate reference genes (*Hc18S rRNA, HcACT7, HcUBC, HcPTB, HcPP2A, HcTUB8, HcMZA, HcRAN*, and *HcEF1*α) were identified from the *H. cannabinus* transcriptome (SRP060459). The full-length cDNA sequences of these genes were cloned from *H. cannabinus* variety Fuhong992, and the sequence information was deposited in GenBank, and the accession numbers were showed in Table 1. Subsequently, specific primers were designed, and the specificity of the primers was confirmed on the basis of the amplification efficiency and specificity: (a) all primers were amplified with a single band after 1.5% agarose gel electrophoresis (Figure S1); (b) a single peak in the melting curve analysis; and (c) the primers amplification efficiency (E) ranged from 97.13 to 118.6%, and the correlation coefficients (R^2^) of the standard curve varied from 0.9949 to 0.9997 (Table 1 and Figure S2).

### Reference gene expression profiles

**Figure 1:**
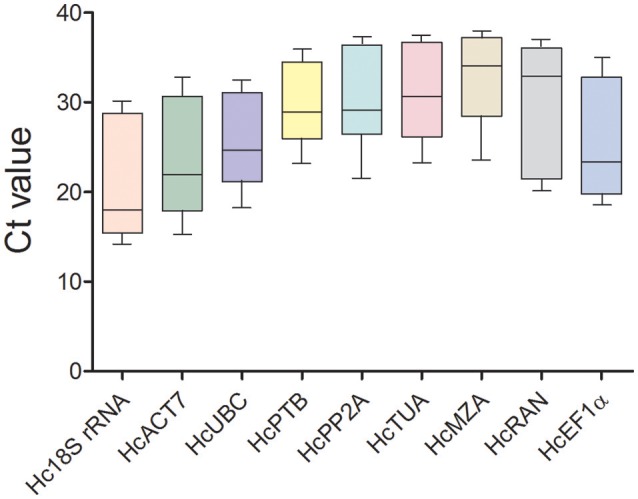
**Ct values of 9 candidate reference genes in all experimental samples**. The box indicates 25th and 75th percentiles. Lines across the box depict the median value. The whiskers represent the 5th and 95th percentiles.

Transcript abundances of 9 candidate reference genes were assessed by qRT-PCR in 396 samples, and the Ct values were examined under three groups including abiotic stresses, hormonal stimuli, and all samples. Lower Ct values represent higher expression abundance, and higher Ct values represent lower expression abundance. As shown in Figure 1 and Table 1, the mean Ct values ranged from 17.98 to 34.04, with SD varied from 1.69 to 2.46 and CV from 4.9 to 12.5% among these 9 candidate genes. These data means that *Hc18S rRNA* expressed abundantly (Ct < 18); *HcACT7, HcUBC*, and *HcEF1*α were moderately expressed genes (21 < Ct < 24); *HcPTB, HcPP2A*, and *HcTUB8* showed slightly lower expression levels (28 < Ct < 31); and *HcRAN* and *HcMZA* showed the lowest expression levels with Ct values as high as 32 and 34 cycles, respectively. Additionally, *HcMZA* showed the least variable gene (CV = 4.9%), followed by *HcPP2A* (6.2%), and *Hc18S rRNA* showed the most variability (12.5%) among the 9 candidate genes. Based on CV values, the stability ranking of all reference genes was as follows: *HcMZA* > *HcPP2A* > *HcPTB* > *HcRAN* > *HcTUB8* > *HcUBC* > *HcEF1*α > *HcACT7* > *Hc18S rRNA* (Table 1). In Brief, these data indicate that transcription levels of reference genes are unstable under different experimental conditions.

### Reference genes stability analysis by geNorm

The geNorm statistical algorithm was used for evaluating expression stability of the 9 candidate references. This algorithm determines the gene expression stability (M) based on the average pairwise variation of all tested genes (Vandesompele et al., 2002; Liu et al., 2016). The lower M means the gene expresses more stable. For kenaf variety Fuhong992, the candidate genes in each experimental group showed much lower M values than the threshold value of 1.5. Therefore, *HcPP2A* and *HcPTB* (*M* = 0.08) were ranked as the most stable, *HcUBC* (*M* = 0.19) was the least gene in the abiotic stress group. Under the hormonal stimuli conditions, *HcACT7* and *Hc18S rRNA* (*M* = 0.10), followed by *HcTUB8* (*M* = 0.11), were identified as the most stable genes, *HcUBC* (*M* = 0.26) showed the worst stability. When all samples was considered, *Hc18S rRNA* and *HcACT7* (*M* = 0.09) featured the most stable genes whereas *HcEF1*α (*M* = 0.21) and *HcUBC* (*M* = 0.25) were the least genes (Table 2). For kenaf varieties Fuhong952, *HcPTB* and *HcEF1*α (*M* = 0.03) were listed as the best genes, *HcUBC* and *HcMZA* were the worst in the abiotic stress group. Under the hormonal stimuli conditions, *HcACT7* and *HcTUB8* (*M* = 0.02), followed by *HcPP2A* (*M* = 0.04), were identified as the most stable genes, while *HcUBC* (*M* = 0.13) gene showed the worst stability. As to total samples, *HcPP2A* and *Hc18S rRNA* (*M* = 0.07) featured the most stable genes whereas *HcTUB8* (*M* = 0.21) was the least (Table S2). For kenaf varieties GV42, *HcPP2A* and *HcACT7* expressed stably, *HcUBC* and *HcEF1*α expressed unstably under abiotic stresses, hormonal stimuli, and total samples. (Table S3).

**Table 2:** **geNorm, NormFinder, BestKeeper and Comprehensive ranking analyzed the gene expression stability in kenaf**.

**Group**	**Rank**	**geNorm**		**NormFinder**		**BestKeeper**			**Comprehensive ranking**	
		**Gene**	**Stability**	**Gene**	**Stability**	**Gene**	**SD**	**CV**	**Gene**	**Geomean value**
Abiotic stress	1	*HcPP2A*	0.08	*HcPP2A*	0.02	*HcMZA*	0.12	0.34	*HcPP2A*	1.26
	2	*HcPTB*	0.08	*HcACT7*	0.04	*HcPP2A*	0.42	1.50	*HcPTB*	2.29
	3	*HcACT7*	0.10	*HcPTB*	0.04	*HcRAN*	0.42	1.91	*HcMZA*	3.11
	4	*Hc18SrRNA*	0.11	*Hc18SrRNA*	0.05	*HcUBC*	0.60	1.85	*HcACT7*	3.17
	5	*HcTUB8*	0.12	*HcTUB8*	0.09	*HcEF1α*	0.70	4.04	*Hc18SrRNA*	3.56
	6	*HcRAN*	0.13	*HcMZA*	0.11	*Hc18SrRNA*	0.96	3.43	*HcRAN*	3.91
	7	*HcMZA*	0.14	*HcRAN*	0.12	*HcPTB*	0.97	4.11	*HcTUB8*	4.82
	8	*HcEF1α*	0.17	*HcEF1α*	0.14	*HcTUB8*	1.49	5.12	*HcEF1α*	5.77
	9	*HcUBC*	0.19	*HcUBC*	0.17	*HcACT7*	1.61	7.52	*HcUBC*	5.81
Hormone stimuli	1	*HcACT7*	0.10	*HcPP2A*	0.03	*HcRAN*	0.37	1.08	*HcPP2A*	1.82
	2	*Hc18SrRNA*	0.10	*HcACT7*	0.03	*HcPP2A*	0.92	5.10	*HcACT7*	1.91
	3	*HcTUB8*	0.11	*HcPTB*	0.04	*HcPTB*	1.03	3.52	*Hc18SrRNA*	2.71
	4	*HcPP2A*	0.12	*HcTUB8*	0.05	*HcMZA*	1.03	3.11	*HcPTB*	2.88
	5	*HcPTB*	0.13	*Hc18SrRNA*	0.07	*HcEF1α*	1.21	5.14	*HcRAN*	2.92
	6	*HcRAN*	0.14	*HcRAN*	0.13	*Hc18SrRNA*	1.26	4.11	*HcTUB8*	3.63
	7	*HcEF1α*	0.17	*HcEF1α*	0.14	*HcUBC*	1.26	4.31	*HcEF1α*	5.24
	8	*HcMZA*	0.21	*HcMZA*	0.23	*HcACT7*	1.41	5.72	*HcMZA*	5.28
	9	*HcUBC*	0.26	*HcUBC*	0.29	*HcTUB8*	1.65	7.58	*HcUBC*	7.27
Total	1	*HcACT7*	0.09	*HcPP2A*	0.03	*HcRAN*	1.19	3.55	*HcPP2A*	2.08
	2	*Hc18SrRNA*	0.09	*HcACT7*	0.04	*HcMZA*	1.21	3.54	*HcACT7*	2.52
	3	*HcTUB8*	0.11	*HcPTB*	0.05	*HcPP2A*	1.33	4.56	*Hc18SrRNA*	2.52
	4	*HcPP2A*	0.12	*Hc18SrRNA*	0.07	*Hc18SrRNA*	1.45	8.09	*HcRAN*	3.11
	5	*HcPTB*	0.13	*HcTUB8*	0.07	*HcEF1α*	1.51	6.47	*HcPTB*	4.16
	6	*HcRAN*	0.15	*HcRAN*	0.14	*HcPTB*	1.58	5.46	*HcTUB8*	4.48
	7	*HcMZA*	0.18	*HcEF1α*	0.15	*HcUBC*	1.72	7.02	*HcMZA*	4.58
	8	*HcEF1α*	0.21	*HcMZA*	0.20	*HcACT7*	1.75	8.04	*HcEF1α*	6.26
	9	*HcUBC*	0.25	*HcUBC*	0.27	*HcTUB8*	1.89	6.19	*HcUBC*	7.96

**Figure 2:**
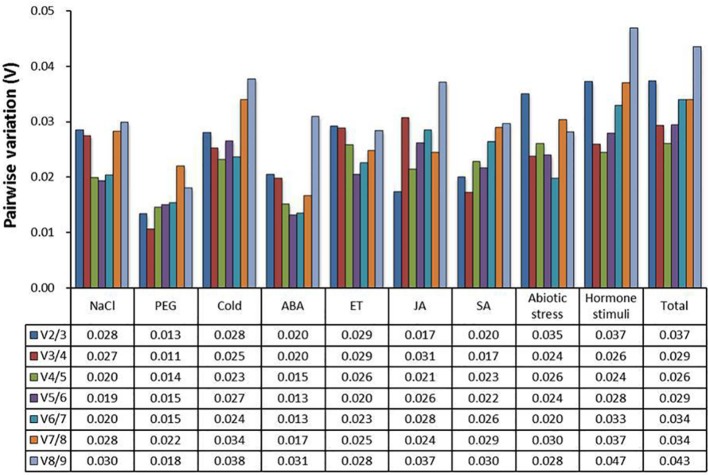
**Pairwise variation (V) of candidate reference genes calculated by geNorm**. Pairwise variation (V_*n*_/V_*n*+1_) was calculated between normalization factors NF_*n*_ and NF_*n*+1_ to determine the optimal number of reference genes. “Abiotic stress” includes NaCl, PEG, and Cold. “Hormone stimuli” includes ABA, ET, JA, and SA. “Total” represents all samples.

The optimal number of reference genes was also determined by geNorm algorithm based on the pairwise variation (V_*n*_) between normalization factors (NF_*n*_). The threshold value (V_*n*_/V_*n*+1_ = 0.15) indicates that the number of reference genes less than or equal to the value of n is sufficient to use as reference gene. As depicted in Figure 2, the pairwise variation value V_2_/V_3_ of all experimental samples was less than 0.15, suggesting that two reference genes should be sufficient for normalization under these conditions.

### Reference genes stability analysis by NormFinder

The NormFinder approach determines the stability of candidate reference genes based on inter- and intra-group variations in expression across different sample groups (Andersen et al., 2004). Similar to the geNorm analysis, the lower M value means the gene expressed more stable. We obtained the similar results through the NormFinder analysis, which predicted *HcPP2A, HcACT7*, and *HcPTB* to be the three stable genes for abiotic stress group, hormonal stimuli group, and the total samples group in Fuhong992 and GV42 (Table 2; Table S3). For kenaf varieties Fuhong952, *HcPP2A, Hc18S rRNA, HcPTB, HcACT7* were the most stable reference genes under different conditions (Table S2). Overall, *HcUBC* and *HcMZA* were ranked as the worst reference gene, which was consistently found in the three subsets.

### Reference genes stability analysis by bestKeeper

The BestKeeper program was applied for evaluating the candidate references stability based on CV and SD of Ct values (Pfaffl et al., 2004). The lower CV ± SD value, the more stable expressed. The analysis results of BestKeeper were also listed in Table 2. For kenaf variety Fuhong992, *HcMZA* (CV ± SD = 0.34 ± 0.12) and *HcPP2A* (CV ± SD = 1.50 ± 0.42) showed remarkably stable expression in the abiotic stress group. *HcRAN* (1.08 ± 0.37) and *HcPP2A* (5.10 ± 0.92) were identified as the two best reference genes in the different hormonal stimuli group. When all samples were analyzed together, the two reference genes, *HcRAN* (3.35 ± 1.19) and *HcMZA* (3.54 ± 1.21), showed the highest expression stabilities in the total group (Table 2). For kenaf variety Fuhong952, *HcRAN* (0.71 ± 0.24) and *HcEF1*α (1.45 ± 0.29) showed stable expression in the abiotic stress group. *HcRAN* (0.32 ± 0.10) and *HcPP2A* (2.36 ± 0.65) were identified as the two best reference genes in hormonal stimuli group. *HcPTB* (2.35 ± 0.64) and *HcPP2A* (2.37 ± 0.66) showed the highest expression stabilities in the total group (Table S2). For kenaf variety GV42, *HcRAN* and *HcTUB8* were the two best reference genes in abiotic stress group, hormonal stimuli group, and total group (Table S3). All these results were inconsistent with those obtained from the geNorm and NormFinder analyses.

### Comprehensive stability analysis

To develop a consistent result, a comprehensive ranking of reference genes was obtained according to the method described by Niu et al. (2015b). The results were shown in Table 2, Tables S2, S3 for Fuhong992, Fuhong952, and GV42, respectively. Based on geNorm algorithm, the best combination of reference gene in each group was further obtained. As shown in Table 3, *HcPP2A* and *HcPTB* were the most stable references under abiotic stress condition; *HcPP2A* and *HcACT7* in the different hormonal stimuli and total samples groups for kenaf variety Fuhong992. For Fuhong952, *HcRAN, HcPP2A*, and *HcPTB* were listed as the most stable genes in the abiotic stress group; *HcPP2A* and *HcACT7* in the different hormonal stimuli group; *HcPP2A* and *HcPTB*, followed by *Hc18S rRNA* in the total samples group. For GV42 variety, *HcPP2A* and *HcACT7* showed the optimal performance in the abiotic stress group, hormonal stimuli group, and the total samples group (Table 3).

**Table 3:** **Suitable reference genes ranked by geNorm and comprehensive analysis in different kenaf varieties**.

**Variety**	**Abiotic stress**		**Hormone stimuli**		**Total**	
	**Most**	**Least**	**Most**	**Least**	**Most**	**Least**
Fuhong992	*HcPP2A*	*HcUBC*	*HcPP2A*	*HcUBC*	*HcPP2A*	*HcUBC*
	*HcPTB*		*HcACT7*		*HcACT7*	
					*Hc18S rRNA*	
Fuhong952	*HcRAN*	*HcMZA*	*HcPP2A*	*HcUBC*	*HcPP2A*	*HcTUB8*
	*HcPP2A*		*HcACT7*		*HcPTB*	
	*HcPTB*				*Hc18S rRNA*	
GV42	*HcPP2A*	*HcEF1α*	*HcPP2A*	*HcUBC*	*HcPP2A*	*HcUBC*
	*HcACT7*		*HcACT7*		*HcACT7*	
					*HcRAN*	

### Validation of the selected reference genes

**Figure 3:**
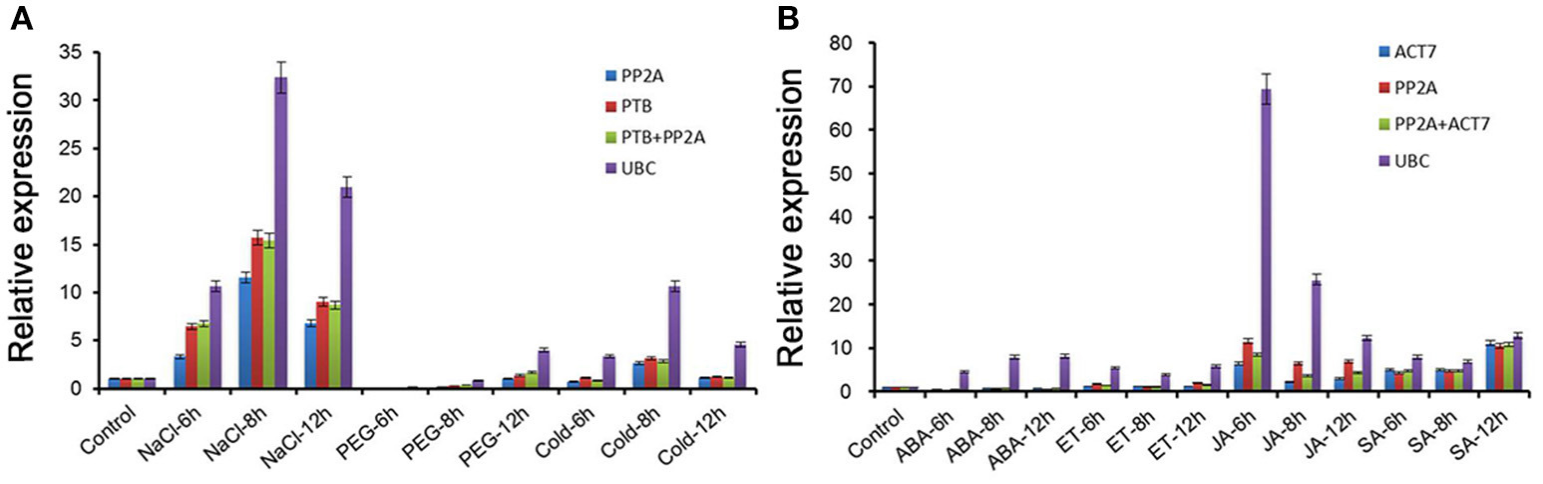
**Relative expression of *HcWRKY28* using the selected reference genes**. Results were normalized using the selected stable references (singly or in combination) and the unstable reference gene in sample sets across **(A)** abiotic stress-, and **(B)** hormonal stimuli-treated samples at 0, 6, 8, 12 h. Bar indicates the standard error (±SE) evaluated from three biological replicates.

To confirm the reliability of the selected references, the relative expression level of *HcWRKY28* was normalized using the most stable and the least stable reference genes by qRT-PCR analysis in kenaf variety Fuhong992. As shown in Figure 3, the relative transcript abundance of *HcWRKY28* under different abiotic stress or hormonal stimuli was found to be impartial when *HcPP2A, HcPTB*, and *HcACT7* or their combinations (*HcPP2A* and *HcPTB* or *HcACT7*) were used as normalization factors. Using references *HcPP2A, HcPTB*, or combination of *HcPP2A* and *HcPTB*, the *HcWRKY28* gene was significantly up-regulated under salinity and cold stress at 8 h, and was down-regulated under drought stress at 6, 8, and 12 h (Figure 3A). Correspondingly, for different hormonal stimuli, the *HcWRKY28* showed relative up-regulation (JA and SA stimuli) or down-regulation (ABA and ET stimuli) at each time point when *HcPP2A, HcACT7* or their combination were used for transcript normalization (Figure 3B). However, when the worst reference gene *HcUBC* used as an internal control, the expression pattern of *HcWRKY28* showed strong fluctuations, and the gene expression change level was 5-fold higher than that by the most stable genes under different abiotic stress and hormonal stimuli (Figure 3).

## Discussion

Agricultural productivity worldwide is challenged by the unfavorable environmental factors, such as drought, salinity and extreme temperatures due to their high intensity of impact and wide distribution (Bartels and Sunkar, 2005; Mittler, 2006). Being sessile, plants have to develop a series of complicated mechanisms to resist and/or adapt to adverse conditions. At the molecular level, transcriptomics and functional genomics approaches have been implemented for understanding the defense mechanisms of plant response to unfavorable environmental conditions. qRT-PCR has become the most powerful method for detection of transcriptomics data and study the molecular mechanisms of plant stress responses. However, accurate gene expression data by qRT-PCR analysis relies on the stable normalization factors. Using a stable reference gene as the normalization factor will ensure the qRT-PCR data to be reliable for target genes. On the contrary, the use of an inappropriate internal reference gene will result in deviation.

To avoid bias introduced, nine commonly used reference genes were cloned from kenaf for evaluation of their expression stability under different stress conditions in the present study. As expected, the distinct stability rankings were generated among the three groups, owing to the different statistical algorithms. For instance, *HcPP2A* and *HcPTB* were ranked as the most stable references in abiotic stress group by geNorm algorithm; *HcPP2A* and *HcPTB*/*HcACT7* by NormFinder; while *HcMZA* and *HcPP2A*/*HcRAN* by BestKeeper. The similar inconsistence was also observed in hormonal stimuli and total groups. To obtain an accordant result, the stability rankings ranked by three algorithms were integrated, generating a comprehensive ranking. It was recommended that *HcPP2A* and *HcACT7* were the top two most stable reference genes in all samples groups. *HcPP2A* also paired with either *HcPTB* or *HcACT7* as the most stable gene by geNorm algorithm across the abiotic stress samples, hormonal stimuli or total samples, respectively. Taken together, our results further confirmed several previous studies, where *PP2A* was also selected as the best normalization factor. For example, *PP2A* expressed stably in *Pennisetum glaucum* under different abiotic stresses and hormonal stimuli (Saha and Blumwald, 2014; Shivhare and Lata, 2016), *Caragana intermedia* under osmotic and heat stress (Zhu et al., 2013), *Agrostis stolonifera* under different abiotic stresses (Chen et al., 2015), *Brassica napus* under drought, salinity and JA stresses (Wang et al., 2014b), *Cynodon dactylon* under different abiotic stresses (Chen et al., 2014). *ACT7*, one of the best reference genes in this study, was also stably expressed under different abiotic stresses and hormonal stimuli of *Daucus carota* (Tian et al., 2015), JA and ABA stresses of *Brassica napus* (Wang et al., 2014b), drought stress of *Populus euphratica* (Wang et al., 2014a), and *Agrostis stolonifera* (Chen et al., 2015). Another stable gene is *PTB*, a novel reference gene identified in *A. thaliana*, has been identified as one of the best reference for *Gossypium hirsutum* (Artico et al., 2010) under abiotic stress. *Hc18S rRNA, HcMZA, HcRAN*, and *HcTUB8* were regarded as unsuitable genes due to their expression variability, thereby rendering them inappropriate to be reference genes. However, good performance of *Hc18SrRNA* under salinity and drought stress conditions was reported in another study of kenaf (Niu et al., 2015a). The possible reason might be that the cold stress samples added and merged into a new stress combination, and then analyzed by different statistical algorithms, finally generating different results. The *MZA* gene showed high stability in *G*. *hirsutum* (Artico et al., 2010), *Phyllostachys edulis* (Fan et al., 2013), and *Solanum lycopersicon* (Exposito-Rodriguez et al., 2008); but low stability in our study though BestKeeper algorithm ranked it 1st position in abiotic stress samples. *RAN* was also the optimal performer in *Cucumis melo* (Kong et al., 2014) treated with growth regulators but was variable under different conditions in the present study. *EF1*α was previously reported as the most stably expressed reference gene in many species, for example, *Glycine max* under drought and salinity stresses (Saraiva et al., 2014), *Solanum tuberosum* under abiotic stresses (Nicot et al., 2005), *Saccharum* spp. under drought and salinity stresses (Guo et al., 2014), *Pennisetum glaucum* under individual and multiple abiotic stresses (Shivhare and Lata, 2016). However, *HcEF1*α was recognized as the least stable gene by the three algorithms, and *UBC*, a commonly used reference gene, was also consistently ranked as the worst gene by geNorm and NormFinder across all samples groups in this study.

To validate the availability of the selected references, the expression levels of *HcWRKY28* gene were normalized by using the selected reference genes. The *HcWRKY28* showed consistent amplification profiles when the two most stable genes were used as internal controls either singly or in combination for both abiotic stresses and hormonal stimuli. While severe disparities occurred when the least stable gene *HcUBC* was used for normalization. These results indicated that *HcPP2A* singly or in combination with *HcACT7* or *HcPTB* are suitable reference gene(s) for transcript normalization under different abiotic stresses, hormonal stimuli and different kenaf varieties. It is without any doubt that the uncharacterized reference genes may also be the better candidates for transcript normalization in kenaf under different conditions. To our knowledge, the present study firstly provides a systematic evaluation on reliability of reference genes used for qRT-PCR normalization in any fiber crop under various abiotic stresses and hormonal stimulus at different stress durations. Therefore, this work will facilitate the accurate qRT-PCR gene expression studies on cross-talking mechanisms between abiotic stresses and hormone signaling pathway in kenaf.

## Author contributions

XN and JQ initiated and designed the research. XN, MC, XH, and HC performed the experiments. XN, AT, and JX analyzed the data. XN, MC, and HC prepared reagents/materials. XN wrote the paper. XN and JQ revised paper.

### Conflict of interest statement

The authors declare that the research was conducted in the absence of any commercial or financial relationships that could be construed as a potential conflict of interest. The reviewer YQ declared a shared affiliation, though no other collaboration, with several of the authors XN, XH, HC, AT, JX, JQ to the handling Editor, who ensured that the process nevertheless met the standards of a fair and objective review.
