# Supplementary material for: Reference Gene Selection for qRT-PCR Normalization Analysis in kenaf (Hibiscus cannabinus L.) under Abiotic Stress and Hormonal Stimuli
Source: Front Plant Sci. 2017 May 12;8:771. doi: 10.3389/fpls.2017.00771 (PMC5427111; doi:10.3389/fpls.2017.00771)
Supplement: Supplementary file 1 [file Presentation1.PDF]

Reference gene selection for qRT-PCR normalization in kenaf (*Hibiscus cannabinus* L.) under abiotic stresses and hormonal stimulus

Xiaoping Niu[1*], Meixia Chen[1, 2], Aifen Tao[1], Jiantang Xu[1], Jianmin Qi[1*]

[1] Key Laboratory for Genetics, Breeding and Multiple Utilization of Crops, Fujian Agriculture and Forestry University, Fuzhou 350002, China

[2] College of Life Sciences, Ningde Normal University, Ningde 350002, China

[*] Corresponding authors. E-mail: qijm863@163.com; xpniu0613@126.com.

Supporting Information

Figure S1. Amplified products of 9 candidate reference genes were separated by 1.5% agarose gel electrophoresis. M: marker 2000.

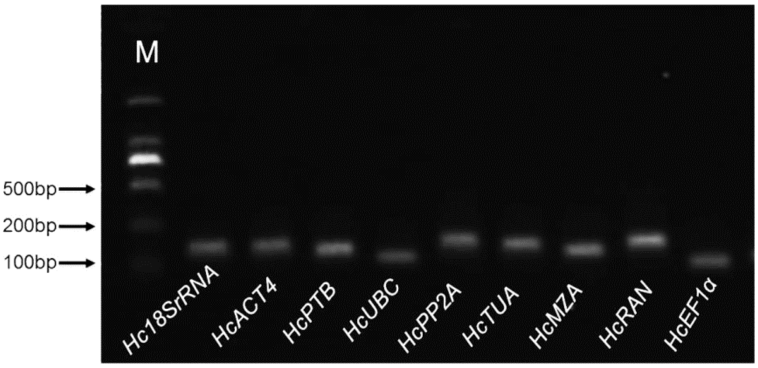

Figure S2. Melting curves of the 9 candidate reference genes tested in this study.

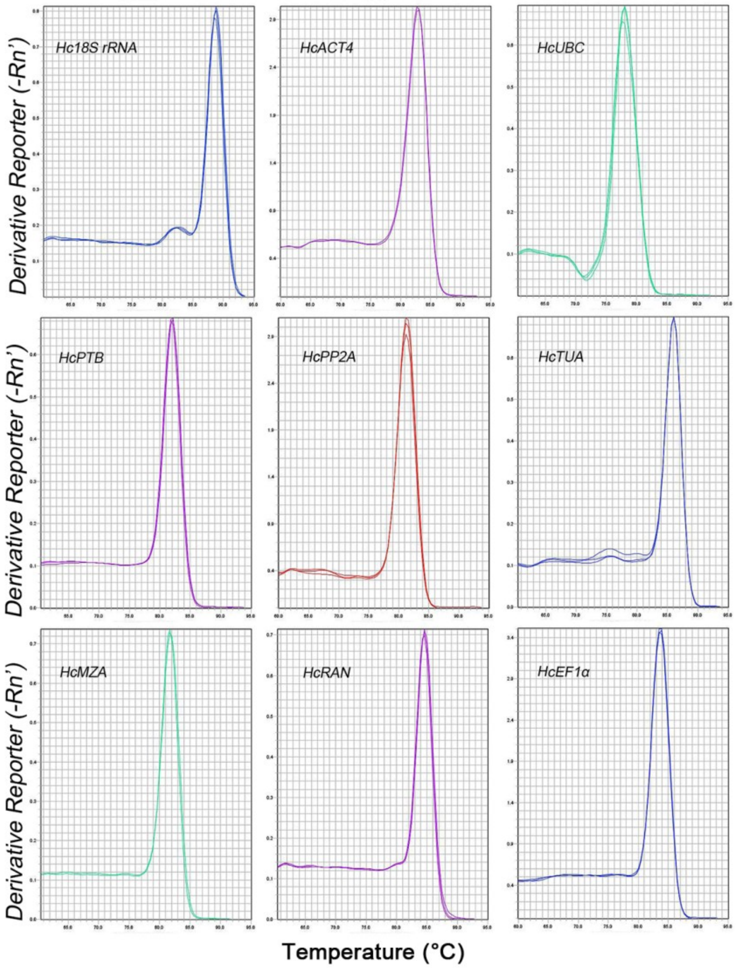

Table S1. Primer sequences for cloning 9 reference genes from *Hibiscus cannabinus*.

Gene	Primer sequence<br>F / R (5'-3')
Hc18S rRNA	ATGAAAAAGGTGGTCGTCTGTTGTC<br>CAGCGGGTAATCCCGCCTGA
HcACT7	ATGGCGGATGCGGAGGATATTCAAC<br>TTAGAAGCACTTCCTGTGGA
HcUBC	ATGGCGTCGAAGCGGATCTTGAAGG<br>TTAAGCATACTTCTGGGTCCAGCTC
HcPTB	ATGGCGGACGCTTCTAAAGTCATTC<br>TTACTGTGAGTTCTCTCTTATTGTC
HcPP2A	ATGGAGATTGATGCAATGGAAGACG<br>TCAGATATGCAAAGCTCTGTAGTGT
HcTUB8	ATGAGAGAAATCATTCACTTACAAA<br>TTCACCTGTATACCAATGCAAGAAG
HcMZA	ATGGCGGGGGCAGCATCTGCACTGT<br>TCAAATAAGTCTTAGTTCGTACTCG
HcRAN	ATGAATCCCGAATATGACTA<br>TAGGAAGAGCAGCAACCACC
HcEF1α	TACCGGTACATCACAGGCTGATGTG<br>TCACTTCTTTTTGGTTGCCTTTTCA

Table S2. Gene expression stability ranked by geNorm, NormFinder, BestKeeper and Comprehensive ranking in Fuhong952.

Group	Rank	geNorm		NormFinder		BestKeeper			Comprehensive Ranking	
		Gene	Stability	Gene	Stability	Gene	SD	CV	Gene	Geomean value
Abiotic stress	1	*HcPTB*	0.03	*HcRAN*	0.02	*HcRAN*	0.24	0.71	*HcRAN*	1.26
	2	*HcEF1α*	0.03	*Hc18SrRNA*	0.02	*HcEF1α*	0.29	1.45	*HcPP2A*	2.41
	3	*HcRAN*	0.06	*HcPP2A*	0.04	*HcUBC*	0.33	1.41	*HcPTB*	2.92
	4	*Hc18SrRNA*	0.06	*HcTUB8*	0.04	*HcPP2A*	0.41	1.44	*Hc18SrRNA*	3.30
	5	*HcTUB8*	0.07	*HcPTB*	0.04	*HcPTB*	0.43	1.55	*HcEF1α*	4.16
	6	*HcACT7*	0.07	*HcACT7*	0.05	*Hc18SrRNA*	0.50	1.86	*HcTUB8*	4.82
	7	*HcPP2A*	0.08	*HcEF1α*	0.05	*HcTUB8*	0.62	1.74	*HcUBC*	5.52
	8	*HcUBC*	0.09	*HcUBC*	0.08	*HcACT7*	0.62	3.25	*HcACT7*	6.21
	9	*HcMZA*	0.10	*HcMZA*	0.09	*HcMZA*	0.69	1.97	*HcMZA*	8.65
Hormone stimuli	1	*HcACT7*	0.02	*HcPP2A*	0.01	*HcRAN*	0.10	0.32	*HcPP2A*	1.59
	2	*HcTUB8*	0.02	*HcACT7*	0.01	*HcPP2A*	0.65	2.36	*HcACT7*	2.52
	3	*HcPP2A*	0.04	*HcTUB8*	0.01	*HcPTB*	0.66	2.45	*HcTUB8*	3.00
	4	*HcPTB*	0.05	*HcPTB*	0.02	*HcMZA*	0.70	2.07	*HcRAN*	3.11
	5	*Hc18SrRNA*	0.05	*Hc18SrRNA*	0.03	*Hc18SrRNA*	0.79	3.04	*HcPTB*	3.30
	6	*HcRAN*	0.06	*HcRAN*	0.06	*HcEF1α*	0.82	3.66	*Hc18SrRNA*	4.64
	7	*HcEF1α*	0.09	*HcEF1α*	0.08	*HcUBC*	0.94	3.95	*HcMZA*	6.07
	8	*HcMZA*	0.10	*HcMZA*	0.12	*HcACT7*	1.06	5.05	*HcEF1α*	6.32
	9	*HcUBC*	0.13	*HcUBC*	0.15	*HcTUB8*	1.28	4.57	*HcUBC*	7.96
Total	1	*HcPP2A*	0.07	*HcPTB*	0.02	*HcPTB*	0.64	2.35	*HcPTB*	1.26
	2	*Hc18SrRNA*	0.07	*Hc18SrRNA*	0.02	*HcPP2A*	0.66	2.37	*HcPP2A*	1.82
	3	*HcPTB*	0.07	*HcPP2A*	0.03	*HcUBC*	0.68	2.85	*Hc18SrRNA*	2.00
	4	*HcACT7*	0.10	*HcACT7*	0.06	*Hc18SrRNA*	0.81	3.08	*HcACT7*	4.16

5	*HcRAN*	0.11	*HcRAN*	0.08	*HcMZA*	1.02	2.97	*HcUBC*	5.01
6	*HcMZA*	0.12	*HcMZA*	0.10	*HcACT7*	1.04	5.16	*HcMZA*	5.31
7	*HcUBC*	0.15	*HcUBC*	0.12	*HcEF1α*	1.08	5.04	*HcRAN*	5.43
8	*HcEF1α*	0.18	*HcEF1α*	0.19	*HcRAN*	1.72	5.33	*HcEF1α*	7.32
9	*HcTUB8*	0.21	*HcTUB8*	0.22	*HcTUB8*	3.83	12.21	*HcTUB8*	8.65

Table S3. Gene expression stability ranked by geNorm, NormFinder, BestKeeper and Comprehensive ranking in GV42.

Group	Rank	geNorm		NormFinder		BestKeeper			Comprehensive Ranking	
		Gene	Stability	Gene	Stability	Gene	SD	CV	Gene	Geomean value
Abiotic stress	1	*HcPP2A*	0.06	*HcPP2A*	0.02	*HcRAN*	0.35	0.99	*HcPP2A*	1.44
	2	*HcACT7*	0.06	*HcACT7*	0.02	*HcTUB8*	0.85	2.45	*HcACT7*	2.52
	3	*HcPTB*	0.07	*HcPTB*	0.02	*HcPP2A*	0.97	3.28	*HcRAN*	3.27
	4	*Hc18SrRNA*	0.09	*Hc18SrRNA*	0.07	*HcMZA*	1.09	3.15	*HcPTB*	3.30
	5	*HcTUB8*	0.12	*HcMZA*	0.11	*Hc18SrRNA*	1.15	6.34	*HcTUB8*	3.63
	6	*HcRAN*	0.13	*HcTUB8*	0.12	*HcPTB*	1.21	4.13	*Hc18SrRNA*	3.91
	7	*HcMZA*	0.14	*HcRAN*	0.13	*HcUBC*	1.27	4.98	*HcMZA*	4.93
	8	*HcUBC*	0.18	*HcUBC*	0.17	*HcACT7*	1.44	6.25	*HcUBC*	7.32
	9	*HcEF1α*	0.21	*HcEF1α*	0.22	*HcEF1α*	1.98	8.10	*HcEF1α*	8.65
Hormone stimuli	1	*HcACT7*	0.05	*HcPP2A*	0.02	*HcRAN*	0.61	1.78	*HcPP2A*	1.44
	2	*HcPP2A*	0.05	*HcACT7*	0.02	*HcTUB8*	0.72	2.05	*HcACT7*	2.62
	3	*HcPTB*	0.06	*HcPTB*	0.03	*HcPP2A*	0.87	3.22	*HcRAN*	3.27
	4	*Hc18SrRNA*	0.07	*Hc18SrRNA*	0.04	*Hc18SrRNA*	0.88	5.54	*HcPTB*	3.30
	5	*HcMZA*	0.08	*HcMZA*	0.06	*HcEF1α*	0.92	4.29	*Hc18SrRNA*	3.63
	6	*HcRAN*	0.09	*HcEF1α*	0.07	*HcPTB*	1.07	4.05	*HcTUB8*	4.58
	7	*HcTUB8*	0.10	*HcRAN*	0.08	*HcUBC*	1.14	5.02	*HcMZA*	5.43
	8	*HcEF1α*	0.11	*HcTUB8*	0.10	*HcMZA*	1.28	3.50	*HcEF1α*	5.94
	9	*HcUBC*	0.14	*HcUBC*	0.15	*HcACT7*	1.38	7.13	*HcUBC*	7.96
Total	1	*HcACT7*	0.05	*HcPP2A*	0.02	*HcRAN*	0.70	2.01	*HcPP2A*	1.71
	2	*HcPP2A*	0.05	*HcACT7*	0.02	*HcTUB8*	0.72	2.06	*HcACT7*	2.62
	3	*HcPTB*	0.07	*HcPTB*	0.04	*Hc18SrRNA*	1.22	7.22	*HcRAN*	3.11
	4	*Hc18SrRNA*	0.10	*Hc18SrRNA*	0.05	*HcMZA*	1.25	3.50	*HcPTB*	3.30

5	*HcTUB8*	0.14	*HcTUB8*	0.14	*HcPP2A*	1.35	4.80	*Hc18SrRNA*	3.30
6	*HcRAN*	0.15	*HcRAN*	0.15	*HcPTB*	1.40	5.07	*HcTUB8*	3.42
7	*HcMZA*	0.17	*HcMZA*	0.18	*HcUBC*	1.42	5.93	*HcMZA*	5.52
8	*HcEF1α*	0.22	*HcEF1α*	0.20	*HcEF1α*	1.60	7.02	*HcEF1α*	7.65
9	*HcUBC*	0.24	*HcUBC*	0.23	*HcACT7*	1.80	8.59	*HcUBC*	7.96
